# Metabolic profiling reveals first evidence of fumigating drug plant *Peganum harmala* in Iron Age Arabia

**DOI:** 10.1038/s42003-025-08096-7

**Published:** 2025-05-23

**Authors:** Barbara Huber, Marta Luciani, Ahmed M. Abualhassan, Daniel Giddings Vassão, Ricardo Fernandes, Thibaut Devièse

**Affiliations:** 1 Max Planck Institute of Geoanthropology, Department of Archaeology, Jena, Germany; 2 Centre de Recherche et d’Enseignement des Géosciences de l’Environnement, Aix-Marseille Université, CNRS, IRD, INRAE, Aix-en-Provence, France; 3 University of Tübingen, Institute for Archaeological Sciences, Tübingen, Germany; 4 University of Vienna, Department of Prehistoric and Historical Archaeology, Vienna, Austria; 5 University of Vienna, Human Evolution and Archaeological Sciences (HEAS), Vienna, Austria; 6 Heritage Commission, Ministry of Culture of the Kingdom of Saudi Arabia, Riyadh, Saudi Arabia; 7 Max Planck Institute for Chemical Ecology, Department of Biochemistry, Jena, Germany; 8 Department of Bioarchaeology, Faculty of Archaeology, University of Warsaw, Warsaw, Poland; 9 Faculty of Arts, Masaryk University, Brno, Czechia; 10 Climate Change and History Research Initiative, Princeton University, Princeton, USA NJ

**Keywords:** Metabolomics, Science in culture

## Abstract

The utilization of medicinal and psychoactive plants in the past represents a pivotal intersection of culture, health, and biodiversity. While such plants in Arabia have been known from classical and medieval textual records, this study provides material evidence of the use of one such plant for fumigation already in the Iron Age. Through metabolic profiling of organic residues recovered from archaeological artefacts at the oasis of Qurayyah, Northwest Arabia, we identified the drug plant *Peganum harmala*. Renowned for its antibacterial, psychoactive and multiple therapeutic properties, its presence highlights the deliberate utilization of local pharmacopeia by ancient communities. This discovery represents not only the first evidence for its use in Iron Age Arabia, but also the most ancient, radiometrically dated material evidence of *Peganum harmala* being used for fumigation globally. Beyond their health benefits, these plants were also valued for their sensory and affective properties. Documenting, understanding and preserving these ancient knowledge systems enriches our understanding of ancient traditions while safeguarding the region’s intangible cultural heritage.

## Abstract (web summary)

Metabolic profiling of Iron Age residues from the oasis of Qurayyah, Northwest Arabia, reveals the earliest material evidence for the fumigation of the drug plant *Peganum harmala*, used in domestic contexts for medicinal and hygienic purposes.

## Introduction

The use of medicinal and psychoactive plants by humans is a tradition with profoundly ancient origins^1–4^. Selecting, processing, and harnessing their bioactive properties marked significant breakthroughs in human history, which laid important foundations for modern pharmacology and traditional medicine^5^. Across cultures, these plants were highly valued for their ability to provide therapeutic benefits and induce altered states of consciousness, playing integral roles in medicinal, sanitary, ritualistic and recreational practices across various cultures^4,6–9^. Through careful observation and experimentation, ancient communities cultivated a deep understanding of their local flora^10,11^. This rich body of knowledge, which included the identification, harvesting, and application of medicinal and psychoactive plants, was refined over generations and passed down through centuries if not longer^11–14^. However, much of the ancient expertise has been lost over time, primarily because these knowledge systems were often transmitted only orally. In contrast to other regions, such as ancient Mesopotamia, Greece, Egypt, or China^11,15,16^, where botanical and medicinal knowledge has been extensively documented also in texts, we lack such ancient written sources from Arabia before the Classical Greek and Islamic periods. Translations of ancient Greek medical texts, such as Galen’s medical works, into Syriac and Arabic occurred only in Late Antiquity^17^.

A promising approach for gaining more information about the use of medicinal and psychoactive plants in earlier periods of history is the analysis of preserved plant residues found in archeological artifacts^7,8,18^. Using techniques such as metabolic profiling, these residues can reveal direct insights into the ancient use of botanical resources, including the identification of the original plant materials, their bioactive properties, and methods of application. This approach offers a unique window into the past, shedding light on the types of plants used for purposes that are often challenging to investigate in archeological contexts, such as medicinal, therapeutic, sensorial, olfactory, sanitary, and recreational practices^7,9,19^. These challenges arise primarily from the limited preservation of these substances, which were often burned during fumigation, directly consumed or processed. In this context, preserved organic residues in archeological artifacts and contexts serve as important archives^18^, offering critical information for plant identification.

**Fig. 1:**
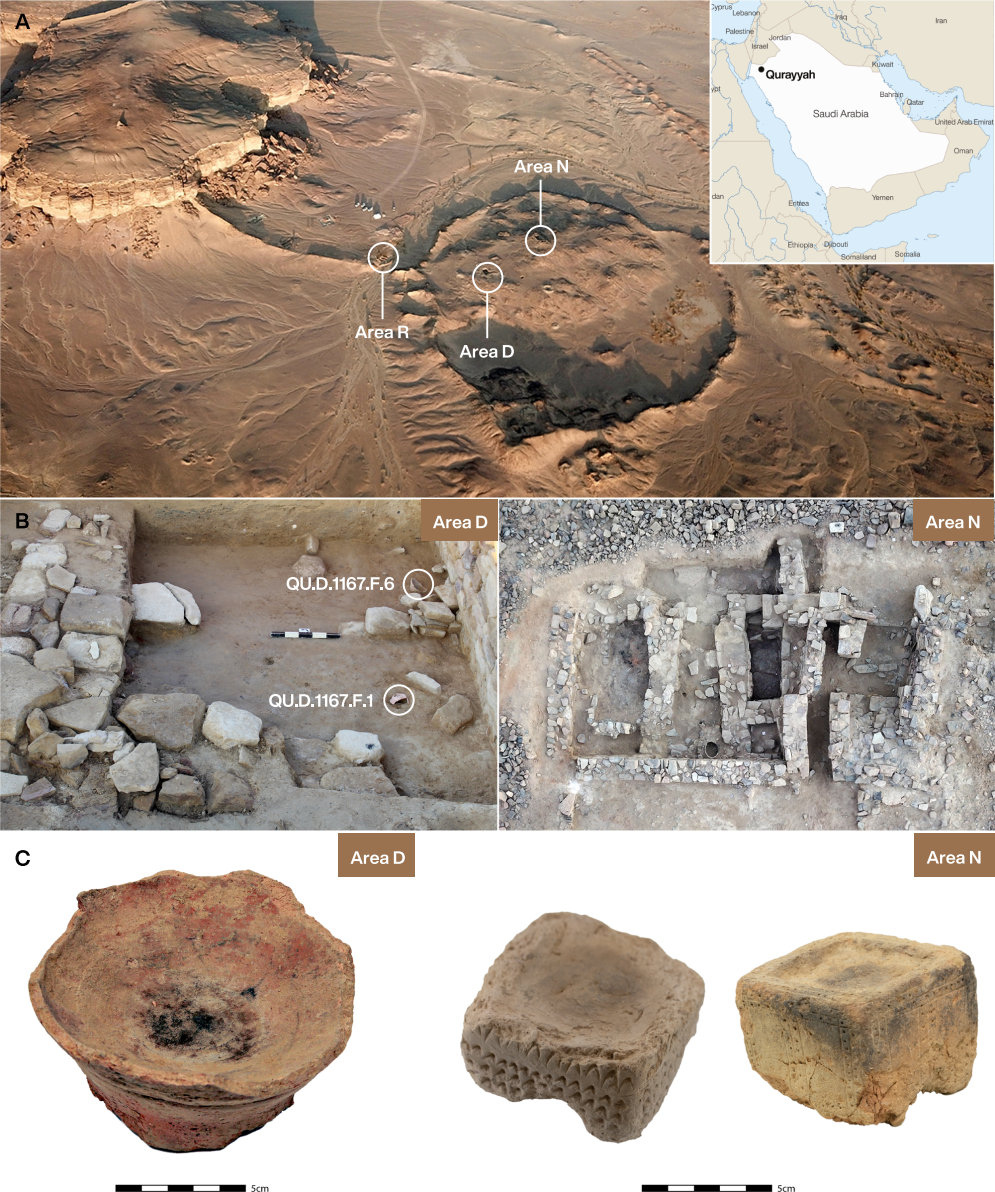
Residential structures and censers from the oasis of Qurayyah. **A** Drone view of Qurayyah with the localization of the excavated Areas D, N, and R, indicated by circles (Photo A. M. Abualhassan). **B** Iron Age residence of Area D with censer QU.D.1167.F.6 and painted vessel QU.D.1167.F.1 in situ (Photo S. McGlone), and Iron Age elite dwelling of Area N (Photo A. M. Abualhassan). **C** Photos of the censer from Area D: QU.D.1167.F.6 and of the two censers from Area N: QU.N.2340.F.3 and QU.N.1253.F.1 (Photos: H. Sell [Area D] and C. Jäger [Area N]). Graphics: Michelle O’Reilly, MPI-GEA.

The present study investigates the use of plant-based substances in Iron Age Arabia, focusing on the archeological site Qurayyah, an ancient oasis settlement in Saudi Arabia (28° 47’ 00” N and 36° 00’ 27” E), which thrived as a significant ‘urban’-like center during the Bronze and Iron Ages (Fig. 1A)^20,21^. This site has yielded numerous censers and fumigation devices from these periods, notable for the preserved organic residues inside them. Although evidence exists for the ancient consumption of drug plants dating back to prehistory in the Americas^8,22–26^, Europe^6,27^, North Africa^9^, and Central Asia^7,28,29^, such practices remain unexplored in pre-classical Arabia, despite the region’s rich diversity of drug plants. Today, among the ca. 2,250 identified plant species in Arabia, nearly 25% are documented for their medicinal uses^13,30^, suggesting that past societies in the region may already have harnessed these plants for their therapeutic and psychoactive properties.

The practice of burning substances for ritual purposes in the oasis of Qurayyah can be traced back to the 3rd millennium BCE, evidenced by a censer found in a final Early Bronze Age grave (2135-1952 cal BCE (IntCal20, 95.4% probability))^31^. Recent excavations have revealed additional fumigation devices from residential areas dating to the Middle Iron Age (first half of 1st millennium BCE; see Supplementary Table 1 for radiocarbon dates). Notably, one such burner (QU.D.1167.F.6) was discovered in Area D, in the courtyard of an Iron Age residence (Fig. 1B). From this object’s inner surface (Fig. 1C), two residue samples (DA-QU_D-1 and DA-QU_D-2) were collected for analysis. Additional devices were discovered in Area N, an elite dwelling of the same age as the Area D residence (Fig. 1B). While one burner (QU.N.1253.F.1) was found in the food production area, another (QU.N.2340.F.3) was discovered in the cellar of the home (see Supplementary Information for additional contextual information). These devices also contained traces of burning and residues on the surface. Sample DA-QU_N-1 was taken from the object in the cooking area, a cuboid burner, which belongs to the building’s latest phase, and sample DA-QU_N-2 was retrieved from the burner in the cellar corresponding to the building’s earliest phase. All censers are made of fired clay.

By employing high-performance liquid chromatography tandem mass spectrometry (HPLC–MS/MS) in multiple reaction monitoring (MRM) mode, we aim to identify the origins of these organic residues and explore their significance in their contexts. MRM is a targeted mass spectrometry technique that facilitates the monitoring of specific precursor and product ion pairs, significantly enhancing the specificity and sensitivity of the analysis. This proves particularly advantageous for detecting compounds present in low concentrations or contained within complex sample matrices, as often encountered in archeological contexts. Here, we present the first material evidence that the plant *Peganum harmala* (commonly known as Syrian Rue, Harmal, or Esfand) was used in fumigation devices. *P. harmala* is known for its antibacterial^32^, psychoactive^33,34^, and multiple therapeutic properties^35,36^, and is widely used in traditional medicine^37,38^. Beyond its well-documented medicinal and psychoactive applications, this discovery invites further exploration of its broader potential uses in ancient Arabian society, including its role in daily life for sanitary purposes, cleansing rituals, and other practical functions.

## Results

### HPLC–MS/MS

**Fig. 2:**
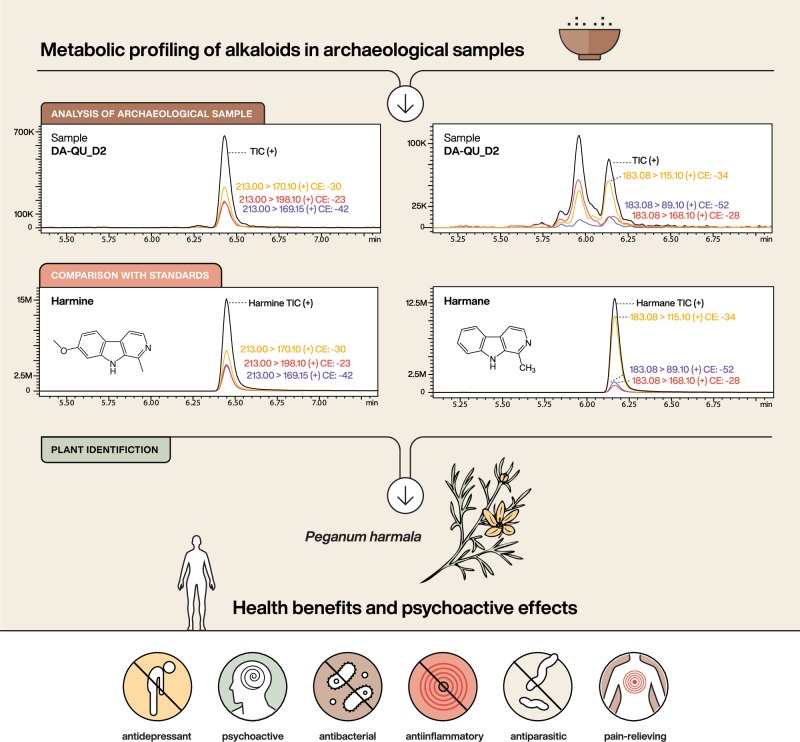
Alkaloids in archeological residues linked to *Peganum harmala* and its psychoactive and therapeutic properties. Archaeological samples (with DA-QU_D2 shown here as an example) were analyzed using targeted metabolomics to detect alkaloids via multiple reaction monitoring (MRM) mode. MRM chromatograms show transitions corresponding to the β-carboline alkaloids harmine and harmane, detected in the archeological sample and compared to analytical standards. For harmine, three transitions are shown: yellow trace (m/z 213.00 → 170.10, collision energy −30 eV), red trace (m/z 213.00 → 198.10, −23 eV), and purple trace (m/z 213.00 → 160.15, −42 eV). For harmane, the following transitions were recorded: yellow trace (m/z 183.08 → 115.10, −34 eV), purple trace (m/z 183.08 → 89.10, −52 eV), and red trace (m/z 183.08 → 168.10, −28 eV). The retention times and transition patterns matched those of reference compounds, confirming the presence of harmine and harmane in the archeological residue with high specificity. The icons below summarize known psychoactive and therapeutic properties of *P. harmala*, including antidepressant, psychoactive, antibacterial, anti-inflammatory, antiparasitic, and pain-relieving effects^32,36,37,39,46,69,75,76^. Graphics: Michelle O’Reilly, MPI-GEA.

The HPLC–MS/MS results show the presence of two tricyclic beta-carboline alkaloids, notably harmine and harmane (Fig. 2; Supplementary Data 1–4), in samples DA-QU_D-1, DA-QU_D-2, and DA-QU_N-1, confirmed through comparison with analytical standards. Harmine and harmane were detected in the archeological sample through specific optimal transitions of precursor and product ions under MRM mode. For harmine, the precursor ion with *m/z* 213.0 fragmented under specific collision energies (CE), producing three product ions: 170.1 at CE: 30 eV, 198.1 at CE: 23 eV, and 169.2 at CE: 42 eV. Similarly, harmane was identified with the precursor ion *m/z* 183.2, which fragmented to yield the product ions 115.1 at CE: 34 eV, 119.1 at CE: 52 eV, and 168.1 at CE: 28 eV (Fig. 2). These transitions, as well as the observed chromatographic retention times, aligned with those obtained from analytical standards, confirming the presence of harmine and harmane in the archeological sample with high specificity. Sample DA-QU_N-2 differed from the other samples as it did not contain these compounds.

These alkaloids occur naturally in species of the *Peganum* genus (Zygophyllaceae family)^35^. These perennial herbaceous shrubs bloom with whitish-yellow flowers and produce globular capsules as fruits, housing blackish seeds^35^. This genus includes four species with a wide distribution, extending from the Mediterranean to East Asia, and even reaching certain areas in America^34^. Specifically, *P. harmala* is widespread across the Mediterranean, Central Asia, North Africa, and Western Asia, including Saudi Arabia, thriving in arid and semi-arid regions^39^. In contrast, *P. nigellastrum* and *P. multisectum* are primarily found in northwestern China, Mongolia, and Russia, while *P. mexicanum* is native to North America^34^. Given that only *P. harmala* is endemic to Saudi Arabia and the other species grow at a considerable geographical distance, *P. harmala* is identified as the most likely and locally available source based on current vegetation. Beyond *P. harmala*, these alkaloids also appear in plants, such as *Passiflora incarnata, Banisteriopsis caapi* (also known as the ayahuasca vine), and other members of the Malpighiaceae family^40^. However, these species are endemic to the Neotropical Region and the Americas, making them unlikely candidates as sources used in Iron Age Arabia. Furthermore, the compound harmane has been identified in tobacco leaves (*Nicotiana* sp.), which are also indigenous to the Americas, as well as in coffee beans, which originated in Africa^41,42^. While the coffee plant (*Coffea sp.)* is currently cultivated in Yemen, its introduction to South Arabia from Ethiopia occurred only in the mid-fifteenth century CE^43^. There is no evidence of coffee cultivation, either in Arabia or Ethiopia, as early as the Iron Age. The chronological gap of more than two millennia between the Iron Age occupation at Qurayyah and the start of coffee cultivation further supports the exclusion of *Coffea sp*. as a potential source. Thus, due to the significant geographical distance and habitat differences of some plant sources and the chronological distance of others, we propose *P. harmala* as the most likely source.

Harmala alkaloids are reversible inhibitors of monoamine oxidase A (MAO-A)^44^. This means that they temporarily inhibit the MAO-A enzyme, which is responsible for breaking down certain neurotransmitters in the brain, such as serotonin and norepinephrine^45^. As a result, these neurotransmitters accumulate to higher levels, stimulating the central nervous system and contributing to the health-benefiting effects of *P. harmala*. Unlike irreversible inhibitors, which permanently deactivate enzymes, harmala alkaloids allow enzyme activity to recover once the compounds are metabolized. Additionally, the broad receptor affinity of harmala alkaloids contributes to their diverse psychopharmacological effects, ranging from sedation to stimulation^46^, as well as exhibiting antibacterial^32^, antiparasitic^36^, and anti-inflammatory activities^39,47^ (Fig. 2). Additionally, they possess immunomodulatory and potential cardiovascular effects, demonstrating a broad spectrum of actions on the human body^48^. However, the effects vary with the dose: small amounts can act as mild and therapeutic stimulants, while larger doses may induce hallucinations, and excessive consumption can even lead to poisoning^49,50^. This dose-dependent response exemplifies the concept of hormesis, where low doses of a substance may trigger beneficial or stimulatory effects, whereas higher doses can lead to adverse or inhibitory outcomes^51^.

In addition to the alkaloid content, samples DA-QU_D-1 and DA-QU_D-2 are characterized by a high abundance of sterol molecules, such as campesterol, β-sitosterol, stigmasterol, and sitostanol, alongside cholesterol (Supplementary Table 2 for further information about lipids). Samples DA-QU_N-1 and DA-QU_N-2 only contained low amounts of β-sitosterol and cholesterol. Phytosterols are widespread among a diverse array of plants^52^, and therefore, undiagnostic for specific plant identification. Common sources are plant oils, seeds, nuts, whole grains, legumes and fruits^53^. Given the high oil content of *P. harmala* seeds, it is plausible that these phytosterols originate from the harmala seeds. Research into *P. harmala* seed oil has revealed anti-inflammatory properties, underscoring the bioactive potential of the oil itself^54^. Nevertheless, the possibility of another plant oil’s contribution cannot be discounted without further evidence.

An additional substance was found in the two burners from Area N. This material was the main substance preserved in sample DA-QU_N-2, which lacked harmala alkaloids. We identified the presence of pentacyclic triterpenoids, specifically α-amyrin and β-amyrin, along with their derivatives (Supplementary Data 3 and 4). These compounds are prevalent across the plant kingdom, notably in plant resins and extracts from the Burseraceae family, which includes species like *Canarium, Protium, Bursera*, and *Commiphora*^55^. Although these compounds alone do not allow for precise species identification, resins from the Burseraceae family are recognized for their beneficial biological activities, including anti-inflammatory, antidepressant, and gastroprotective effects^55–57^. In sample DA-QU_N-1, these compounds co-occurred with beta-carboline alkaloids, suggesting a possible mixture of two substances. Alternatively, the two substances might have been used sequentially within the same fumigation device. The presence of mixtures of substances is known from the oasis of Tayma, another Northwest Arabian oasis settlement 280 km south-east of Qurayyah, where censers from Iron Age graves also revealed residues containing amyrin compounds mixed with conifer and *Pistacia* resins^19,58^. Additionally, at Madaʾin Salih, a settlement part of the oasis of al-ʿUla, 300 km south of Qurayyah, amyrin compounds were recovered from textiles used in Nabataean burial practices^59^. They were interpreted as constituents of elemi resins (*Canarium* sp.). However, there is no evidence of *P. harmala* or other drug plant use from settlement contexts in either Tayma or in Madaʾin Salih, thus corroborating a pattern of use of *P. harmala* in settlement contexts and not in funerary ones. The several censers from funerary contexts in Qurayyah (Area R) have not been analyzed yet.

## Discussion

The metabolic analysis of the alkaloid content in the samples provides the most ancient, radiometrically dated, material evidence of *P. harmala* in fumigation devices. *P. harmala* was traditionally consumed in two main forms: by ingestion, often prepared as a tea or concoction, and through the inhalation of smoke from burning its seeds and roots^1,9,60^. Occasionally, it has been reported for external application, e.g., to treat skin conditions^61,62^. Our case study specifically highlights the practice of burning, as the residues were recovered from fumigation devices at the oasis of Qurayyah, setting it apart from ingestion-based practices and topical treatments. To our knowledge, this represents the earliest material evidence for *P. harmala*’ use as a substance to be burnt in censers in the past. In contrast, earlier documented cases, such as seeds of *P. harmala* found at the Predynastic site of Maadi, Egypt, only confirm the plant’s presence but provide no evidence of its specific applications^63^. Additionally, the archeological context at Qurayyah reveals that the fumigation devices containing harmala alkaloids were recovered exclusively from residential areas. *P. harmala* was used within dwellings, indicating its role at the oasis was most likely for domestic purposes connected to the household rather than for public ceremonies or funerary rituals.

While we have identified the original plant material, its bioactive properties, the method of application, and the context of use, the specific purposes for burning *P. harmala* at Qurayyah may have been multiple. Drawing on its historical and traditional use in other regions, one plausible hypothesis is its use for medicinal and therapeutic purposes, given its well-documented health benefits^34^. Today, the plant is still part of ethnomedicine in Saudi Arabia, where reliance on medicinal plants continues to be a common and valued practice^13^. In traditional medicine, particularly in West Asian and North African systems, *P. harmala* seeds have been recognized for a variety of properties, including analgesic, antithrombotic, carminative, anthelmintic, anti-inflammatory, galactagogue, and emmenagogue effects^35,38^. The administration of *P. harmala* is used to manage conditions such as joint pain, chronic headache, toothache, and rheumatoid arthritis^64^. Other applications described in traditional medicine include its use as a sedative for alleviating nervousness, as well as its potential roles as an antidepressant and mood stabilizer^44,65^. Concerning women’s health, it has been reported as an abortifacient, as well as for regulating menstrual flow and promoting or increasing breast milk production^66,67^. Also in ancient Greek texts, the plant is recognized for its medicinal value, being utilized as a vermifuge to expel tapeworms and as a treatment for fevers^68^.

It is plausible that, beyond human health applications, these plants may have also been used for veterinary purposes. *P. harmala*’s known sedative effects when consumed by farm animals^69,70^ could suggest a dual use in managing human and animal health at the oasis settlement. However, the method of application—fumigation—argues against this, as the plant was typically ingested by animals rather than inhaled as smoke. Similarly, for some human health benefits, the substance is more commonly consumed as a beverage or applied topically. Nevertheless, there are applications in traditional medicine where the burning and inhalation of *P. harmala* seeds was used, for instance, to relieve toothaches and headaches, for anti-arthritic and anti-inflammatory purposes, as a mood stabilizer, and generally to relieve pain^38,64^.

Beyond its therapeutic applications, *P. harmala* was documented in current traditional fumigation practices, particularly in ritualistic and spiritual contexts where its psychoactive properties were harnessed to induce altered states of consciousness^60^. At high doses, harmala alkaloids can produce intense hallucinations, euphoria, and other central nervous system effects^46^. The use of *P. harmala* at the oasis settlement could, therefore, suggest intentional exposure to its psychoactive effects. However, in order to have a hallucinogenic-like effect^71^, it must have been consumed in high concentrations, but the excessive consumption of *P. harmala* as a recreational psychoactive agent can be toxic, with several cases of harmala-related poisoning through overdosing already reported^50^. Beyond dosage, the modality of administration is crucial as well, where fumigation differs from oral intake, as inhalation delivers the active compounds through the respiratory system, potentially altering their absorption, distribution, and impact on the body. Another ritualistic and purifying purpose attested in modern-day Iran is the burning of *P. harmala* seeds to ward off evil.

In connection with purifying and cleansing rituals and routines, the practical applications of *P. harmala* must also be considered, particularly its potential use for sanitary and hygienic purposes. Due to its antibacterial and antifungal properties, the smoke produced from burning *P. harmala* seeds was traditionally used as a disinfectant agent to cleanse spaces and reduce the spread of illnesses^72^. At an oasis settlement like Qurayyah, maintaining hygienic habits in daily life would have been essential for minimizing health risks. In this context, *P. harmala* smoke may have played a role in air purification and disinfecting living spaces. Its practical properties further extend to its use as an insect repellent, offering protection against pests in domestic contexts—a significant concern in warm, oasis environments. In the context of air purification, the use of *P. harmala* primarily for its fragrance is unlikely because *P. harmala* smoke has a rather pungent scent, making it less suitable for olfactory purposes. Parallels from other northwest Arabian oases in the Iron Age indicate that other aromatic substances, such as *Commiphora* and coniferous resins, as well as *Pistacia* were commonly used for incense burning^19,58^.

Given its repeated association with households and its absence from tombs and temples in other oases (Tayma, al-ʿUla^58,59^), the most likely purpose of fumigating *P. harmala* at the oasis settlement of Qurayyah appears to be primarily medicinal or practical in nature. The plant’s documented properties suggest its use for sanitary and hygienic purposes, such as air purification, disinfection, and pest control, which would have been particularly relevant in a domestic oasis environment. Additionally, traditional medicinal practices, both historical and modern, attribute *P. harmala* a range of therapeutic benefits, making it plausible that its smoke was used to alleviate ailments and all sorts of pains. While the plant’s psychoactive potential cannot be entirely dismissed, achieving hallucinogenic effects would have required significantly higher doses, which seems unlikely in this context. Therefore, the use of *P. harmala* at Qurayyah may reflect a combination of practical hygienic applications and medicinal fumigation for treating health conditions within a domestic setting.

The evidence for the burning of *P. harmala* at Qurayyah as early as the Middle Iron Age—approximately 2700 years ago—underscores the deep historical roots of this traditional use of native plants. This discovery not only revives knowledge of ancient practices and highlights a longstanding legacy of medicinal plant use but also contributes to safeguarding the region’s intangible cultural heritage. At the same time, it is equally important to protect and preserve the traditional knowledge that still exists today. In Arabia, traditional plant-based remedies remain deeply valued within their communities^13^. However, such practices are increasingly disappearing. This underscores the urgent need to document and preserve this rich ethnobotanical knowledge before it is lost entirely, along with its historical context. Furthermore, utilizing the information stored in ancient organic remains could enable the recovery of bioactive compounds that have been forgotten over time^73^, potentially leading to the development of innovative plant-based therapies.

## Methods

### Sampling

The organic residue samples come from the archeological site Qurayyah in Saudi Arabia. The excavation permit for research in Qurayyah, as well as for analysis, was issued by the former Saudi Commission for Tourism and Antiquities (now Heritage Commission of the Ministry of Culture). Sampling of organic residues from the incense burners located in Area N was conducted at the archeological site post-excavation, while the samples from Area D were taken within the laboratories of the Max Planck Institute of Geoantropology in Jena, Germany, following established protocols^19,52^. Samples were exported in full accordance with relevant permits and local laws. Prior to sampling, the uppermost inner layer of each burner was meticulously abraded to eliminate potential surface contaminants. The prepared sampling spots then underwent targeted sampling, where visible incrustations were excised using a scalpel, and deeper matrix penetration was achieved by employing a Dremel 200 drill outfitted with a tungsten carbide abrasive bit to extract approximately 2 g of powder from the residual compounds absorbed into the clay matrix. Drill bits were rigorously cleansed with methanol between samplings to preclude any cross-contamination. Typically, a 1 × 1 cm section was drilled to a depth of 2–3 mm. The resultant powder was collected on sterile aluminum foil before being transferred into pre-cleaned glass vials for subsequent analysis.

### Materials

HPLC grade methanol (MeOH) and Dichloromethane (DCM) were obtained from Sigma-Aldrich (Munich, Germany), acetonitrile (ACN) and ultrapure water from Biosolve (Valkenswaard, Netherlands), and formic acid (FA) from VWR (Leuven, Belgium). The analytical standards α- and β-amyrin, cholesterol, campesterol, β-sitosterol, stigmasterol, harmane, and harmine were purchased from Sigma-Aldrich (Munich, Germany), and sitostanol from Avanti (Darmstadt, Germany).

### Extraction and analysis

Plant secondary metabolites and lipids were isolated from ancient organic residues by pressurized solvent extraction (PSE), adhering to previously established protocols^74^. The extraction was facilitated by a Büchi E-916 Pressurized Speed Extractor, which utilized high temperatures and pressures to optimize the recovery of organic compounds. Prior to extraction, samples were homogenized to a uniform particle size using a mortar and pestle. These homogenized samples were then combined with quartz sand (Büchi, 0.3–0.9 mm) at an approximate ratio of 1:5 (sample to sand) and transferred to stainless steel extraction cells positioned within the PSE device’s heating block. The samples were extracted using DCM and MeOH (2:1, v/v) over three extraction cycles, each comprising a 1-min heat-up phase, a 15-min hold, and a 2-min discard interval. Conditions were set at 50 °C and 100 bar. The resultant extracts were collected in glass vials capped with Teflon septa. These extracts were then concentrated to about 1 mL via rotary evaporation. Aliquots of the concentrated extracts were evaporated and resuspended in HPLC-grade methanol.

HPLC–MS/MS analyses were conducted using a Shimadzu LCMS-8050 triple-quadrupole system with an electrospray ionization (ESI) source. The HPLC setup included LC-30AD binary pumps, a DGU-20A5R solvent degasser, CTO-20AC column oven, and a SIL-30AC auto sampler. Analytes were separated on two different columns: a Shimadzu Shimpack Velox SP-C18 and a Restek Raptor Biphenyl, both 100 mm × 2.1 mm with a 2.7 µm particle size. The samples were run with a gradient mobile phase of A, H_2_O:0.1% FA, and B, ACN, in duplicates with blanks in between. A consistent column temperature of 25 °C was maintained throughout the gradient program, which started with 0.5% B for the first minute, increased to 80% B at 10 min, reached 100% B at 15 min with a hold until 17.5 min, and then reverted to 0.5% B, holding until 20 min. Flow rates were adjusted to 0.2 mL/min for the C18 column and 0.3 mL/min for the biphenyl column. Injection volumes of 1 or 2 µL based on sample concentration were injected onto the system and analyzed in positive and negative ESI mode.

Data collection and processing were conducted using LabSolutions software (Shimadzu, Kyoto, Japan), which also facilitated the optimization of MRM mode parameters for the targeted compounds. Authentic analytical standards were employed to optimize the MRM parameters, essential for screening specific compounds in archeological samples. These parameters included precursor and product *m/z*, dwell times, collision energy and Q1 and Q3 pre-bias voltages (refer to Supplementary Data 5 for detailed MRM parameters).

### Reporting summary

Further information on research design is available in the Nature Portfolio Reporting Summary linked to this article.

## Supplementary information

Supplementary Information

Description of Additional Supplementary Files

Supplementary Data 1-4

Supplementary Data 5

Reporting summary

## Data Availability

All data generated or analyzed during this study are included in this article and its supplementary information files. For any additional information, please contact Barbara Huber (huber@gea.mpg.de) or Marta Luciani (marta.luciani@univie.ac.at).

## References

[1] Samorini, G. The oldest archeological data evidencing the relationship of Homo sapiens with psychoactive plants: a worldwide overview. *J. Psychedelic Stud.* **3**, 63–80 (2019).

[2] Guerra-Doce, E. Psychoactive substances in prehistoric times: examining the archaeological evidence. *Time Mind* **8**, 91–112 (2015).

[3] *Consuming Habits: Drugs in History and Anthropology*. (Routledge, London, 1996).

[4] Hardy, K. Plant use in the lower and middle palaeolithic: food, medicine and raw materials. *Quat. Sci. Rev.* **191**, 393–405 (2018).

[5] Nasim, N., Sandeep, I. S. & Mohanty, S. Plant-derived natural products for drug discovery: current approaches and prospects. *Nucleus* **65**, 399–411 (2022). 10.1007/s13237-022-00405-3 PMC9579558 36276225

[6] Guerra-Doce, E. et al. Direct evidence of the use of multiple drugs in Bronze Age Menorca (Western Mediterranean) from human hair analysis. *Sci. Rep.* **13**, 4782 (2023). 37024524 10.1038/s41598-023-31064-2 PMC10079862

[7] Ren, M. et al. The origins of cannabis smoking: chemical residue evidence from the first millennium BCE in the Pamirs. *Sci. Adv.* **5**, eaaw1391 (2019). 31206023 10.1126/sciadv.aaw1391 PMC6561734

[8] Robinson, D. W. et al. *Datura* quids at Pinwheel Cave, California, provide unambiguous confirmation of the ingestion of hallucinogens at a rock art site. *Proc. Natl. Acad. Sci. USA* **117**, 31026–31037 (2020). 33229522 10.1073/pnas.2014529117 PMC7733795

[9] Tanasi, D. et al. Multianalytical investigation reveals psychotropic substances in a ptolemaic Egyptian vase. *Sci. Rep.* **14**, 27891 (2024). 39537764 10.1038/s41598-024-78721-8 PMC11561246

[10] Borchardt, J. K. The beginnings of drug therapy: ancient mesopotamian medicine. *Drug N. Perspect.* **15**, 187 (2002). 10.1358/dnp.2002.15.3.840015 12677263

[11] Geller, M. J. *Ancient Babylonian Medicine*. (Wiley-Blackwell, Oxford, UK, 2010). 10.1002/9781444319996.

[12] Aziz, M. A., Khan, A. H., Adnan, M. & Ullah, H. Traditional uses of medicinal plants used by Indigenous communities for veterinary practices at Bajaur Agency, Pakistan. *J. Ethnobiol. Ethnomed.* **14**, 11 (2018). 29378636 10.1186/s13002-018-0212-0 PMC5789696

[13] Aati, H., El-Gamal, A., Shaheen, H. & Kayser, O. Traditional use of ethnomedicinal native plants in the Kingdom of Saudi Arabia. *J. Ethnobiol. Ethnomed.* **15**, 2 (2019). 30626417 10.1186/s13002-018-0263-2 PMC6325684

[14] Manzoor, M. et al. The local medicinal plant knowledge in Kashmir Western Himalaya: a way to foster ecological transition via community-centred health seeking strategies. *J. Ethnobiol. Ethnomed.* **19**, 56 (2023). 38037066 10.1186/s13002-023-00631-2 PMC10688143

[15] Cai, J. & Zhen, Y. Medicine in Ancient China. in *Medicine Across Cultures* (ed Selin, H.) vol. 3 49–73 (Kluwer Academic Publishers, Dordrecht, 2003).

[16] Nunn, J. F. *Ancient Egyptian Medicine*. (University of Oklahoma Press, 2002).

[17] Kessel, G. Syriac Medicine. in *The Syriac world* (ed King, D. H.) 438–459 (Routledge, London, 2019).

[18] Huber, B., Larsen, T., Spengler, R. N. & Boivin, N. How to use modern science to reconstruct ancient scents. *Nat. Hum. Behav.* **6**, 611–614 (2022). 35347242 10.1038/s41562-022-01325-7

[19] Huber, B. Incense burners at the Oasis of Tayma, northwest Arabia: an olfactory perspective. *Pol. Archaeol. Mediterr.*10.31338/uw.2083-537X.pam29.1.14 (2020).

[20] Luciani, M. On the Formation of ‘Urban’ Oases in Arabia: New Perspectives from the North-west. in *The Archaeology of the Arabian Peninsula 2*: *Connecting the Evidence. Proceedings of the International Workshop held at the 10th International Congress on the Archaeology of the Ancient Near East in Vienna on April 25, 2016 (OREA;* Vol. 19) (ed Luciani, M.) 89–119 (Austrian Academy of Sciences Press, Vienna, 2021).

[21] Luciani, M. Qurayyah. in *Thematic Dictionary of Ancient Arabia* (Thematic Dictionary of Ancient Arabia, 2023). 10.60667/TDAA-0138.

[22] Boyd, C. E. & Dering, J. P. Medicinal and hallucinogenic plants identified in the sediments and pictographs of the Lower Pecos, Texas Archaic. *Antiquity* **70**, 256–275 (1996).

[23] El-Seedi, H. R., Smet, P. A. G. M. D., Beck, O., Possnert, G. & Bruhn, J. G. Prehistoric peyote use: alkaloid analysis and radiocarbon dating of archaeological specimens of Lophophora from Texas. *J. Ethnopharmacol.* **101**, 238–242 (2005). 15990261 10.1016/j.jep.2005.04.022

[24] Zimmermann, M. et al. Metabolomics-based analysis of miniature flask contents identifies tobacco mixture use among the ancient Maya. *Sci. Rep.* **11**, 1590 (2021). 33452410 10.1038/s41598-021-81158-y PMC7810889

[25] Adovasio, J. M. & Fry, G. F. Prehistoric psychotropic drug use in Northeastern Mexico and Trans-Pecos Texas. *Econ. Bot.* **30**, 94–96 (1976).

[26] Dillehay, T. D. et al. Early Holocene coca chewing in northern Peru. *Antiquity* **84**, 939–953 (2010).

[27] Askitopoulou, H., Ramoutsaki, I. A. & Konsolaki, E. Archaeological evidence on the use of opium in the Minoan world. *Int. Congr. Ser.* **1242**, 23–29 (2002).

[28] Guerra-Doce, E. The origins of inebriation: archaeological evidence of the consumption of fermented beverages and drugs in prehistoric Eurasia. *J. Archaeol. Method Theory* **22**, 751–782 (2015).

[29] Long, T., Wagner, M., Demske, D., Leipe, C. & Tarasov, P. E. Cannabis in Eurasia: origin of human use and Bronze Age trans-continental connections. *Veget Hist. Archaeobot.* **26**, 245–258 (2017).

[30] El-Seedi, H. R. et al. Saudi Arabian plants: a powerful weapon against a plethora of diseases. *Plants* **11**, 3436 (2022). 36559548 10.3390/plants11243436 PMC9783889

[31] Luciani, M. Area B. in *Qurayyah 2017. Report on the Third Season of the Joint Saudi Arabian-Austrian Archaeological Project* (eds Luciani, M. & Asiri, R.) vol. 31 33–36 (2022).

[32] Apostolico, I. et al. Chemical composition, antibacterial and phytotoxic activities of *Peganum harmala* seed essential oils from five different localities in Northern Africa. *Molecules* **21**, 1235 (2016). 27649128 10.3390/molecules21091235 PMC6273081

[33] Moshiri, M., Etemad, L., Soheila, J. & Alizadeh, A. *Peganum harmala* intoxication, a case report. *Avicenna J. Phytomed.* **3**, 288–292 (2013). 25050285 PMC4075715

[34] Sharifi-Rad, J. et al. Peganum spp.: a comprehensive review on bioactivities and health-enhancing effects and their potential for the formulation of functional foods and pharmaceutical drugs. *Oxid. Med. Cell. Longev.* **2021**, 1–20 (2021). 10.1155/2021/5900422 PMC8260309 34257813

[35] Shahrajabian, M. H., Sun, W. & Cheng, Q. Improving health benefits with considering traditional and modern health benefits of *Peganum harmala*. *Clin. Phytosci.* **7**, 18 (2021).

[36] Zhu, Z., Zhao, S. & Wang, C. Antibacterial, antifungal, antiviral, and antiparasitic activities of *Peganum harmala* and its ingredients: a review. *Molecules* **27**, 4161 (2022). 35807407 10.3390/molecules27134161 PMC9268262

[37] Moloudizargari, M., Mikaili, P., Aghajanshakeri, S., Asghari, M. & Shayegh, J. Pharmacological and therapeutic effects of *Peganum harmala* and its main alkaloids. *Phcogn. Rev.* **7**, 199 (2013). 10.4103/0973-7847.120524 PMC3841998 24347928

[38] Niroumand, M. C., Farzaei, M. H. & Amin, G. Medicinal properties of *Peganum harmala* L. in traditional Iranian medicine and modern phytotherapy: a review. *J. Tradit. Chin. Med.* **35**, 104–109 (2015). 25842736 10.1016/s0254-6272(15)30016-9

[39] Abbas, M. W. et al. Antioxidant and anti-inflammatory effects of *Peganum harmala* extracts: an in vitro and in vivo study. *Molecules* **26**, 6084 (2021). 34641627 10.3390/molecules26196084 PMC8512429

[40] *Meyler’s Side Effects of Drugs: The International Encyclopedia of Adverse Drug Reactions and Interactions*. (Elsevier, Amsterdam Boston Heidelberg, 2016).

[41] Herraiz, T. & Chaparro, C. Human monoamine oxidase enzyme inhibition by coffee and β-carbolines norharman and harman isolated from coffee. *Life Sci.* **78**, 795–802 (2006). 16139309 10.1016/j.lfs.2005.05.074

[42] Poindexter, E. H. & Carpenter, R. D. The isolation of harmane and norharmane from tobacco and cigarette smoke. *Phytochemistry* **1**, 215–221 (1962).

[43] Montagnon, C., Sheibani, F., Benti, T., Daniel, D. & Bote, A. D. Deciphering early movements and domestication of *Coffea arabica* through a comprehensive genetic diversity study covering Ethiopia and Yemen. *Agronomy* **12**, 3203 (2022).

[44] Herraiz, T. & Guillén, H. Monoamine oxidase-A inhibition and associated antioxidant activity in plant extracts with potential antidepressant actions. *BioMed. Res. Int.* **2018**, 1–10 (2018). 10.1155/2018/4810394 PMC5820675 29568754

[45] Berlowitz, I., Egger, K. & Cumming, P. Monoamine oxidase inhibition by plant-derived β-carbolines; implications for the psychopharmacology of tobacco and ayahuasca. *Front. Pharmacol.* **13**, 886408 (2022). 35600851 10.3389/fphar.2022.886408 PMC9121195

[46] Herraiz, T., González, D., Ancín-Azpilicueta, C., Arán, V. J. & Guillén, H. β-Carboline alkaloids in *Peganum harmala* and inhibition of human monoamine oxidase (MAO). *Food Chem. Toxicol.* **48**, 839–845 (2010). 20036304 10.1016/j.fct.2009.12.019

[47] Patel, K., Gadewar, M., Tripathi, R., Prasad, S. & Patel, D. K. A review on medicinal importance, pharmacological activity and bioanalytical aspects of beta-carboline alkaloid “Harmine”. *Asian Pac. J. Trop. Biomed.* **2**, 660–664 (2012). 23569990 10.1016/S2221-1691(12)60116-6 PMC3609365

[48] Shi, C.-C., Chen, S.-Y., Wang, G.-J., Liao, J.-F. & Chen, C.-F. Vasorelaxant effect of harman. *Eur. J. Pharmacol.* **390**, 319–325 (2000). 10708740 10.1016/s0014-2999(99)00928-0

[49] Kartal, M., Altun, M. L. & Kurucu, S. HPLC method for the analysis of harmol, harmalol, harmine and harmaline in the seeds of *Peganum harmala* L. *J. Pharm. Biomed. Anal.* **31**, 263–269 (2003). 12609665 10.1016/s0731-7085(02)00568-x

[50] Yuruktumen, A., Karaduman, S., Bengi, F. & Fowler, J. Syrian rue tea: A recipe for disaster. *Clin. Toxicol.* **46**, 749–752 (2008). 10.1080/15563650701323205 18803088

[51] Wan, Y. et al. Current advances and future trends of hormesis in disease. *npj Aging* **10**, 26 (2024). 38750132 10.1038/s41514-024-00155-3 PMC11096327

[52] Huber, B. et al. Biomolecular characterization of 3500-year-old ancient Egyptian mummification balms from the Valley of the Kings. *Sci. Rep.* **13**, 12477 (2023). 37652925 10.1038/s41598-023-39393-y PMC10471619

[53] Salehi, B. et al. Phytosterols: from preclinical evidence to potential clinical applications. *Front. Pharmacol.* **11**, 599959 (2021). 33519459 10.3389/fphar.2020.599959 PMC7841260

[54] Khadhr, M. et al. HPLC and GC–MS analysis of tunisian *Peganum harmala* seeds oil and evaluation of some biological activities. *Am. J. Therap.* **24**, e706–e712 (2017). 27058575 10.1097/MJT.0000000000000402

[55] Thirupathi, A., Silveira, P., Nesi, R. & Pinho, R. β-Amyrin, a pentacyclic triterpene, exhibits anti-fibrotic, anti-inflammatory, and anti-apoptotic effects on dimethyl nitrosamine–induced hepatic fibrosis in male rats. *Hum. Exp. Toxicol.* **36**, 113–122 (2017). 27009110 10.1177/0960327116638727

[56] Oliveira, F. A. et al. Protective effect of α- and β-amyrin, a triterpene mixture from Protium heptaphyllum (Aubl.) March. Trunk wood resin, against acetaminophen-induced liver injury in mice. *J. Ethnopharmacol.* **98**, 103–108 (2005). 15763370 10.1016/j.jep.2005.01.036

[57] Oliveira, F. Gastroprotective and anti-inflammatory effects of resin from *Protium heptaphyllum* in mice and rats. *Pharmacol. Res.* **49**, 105–111 (2004). 14643690 10.1016/j.phrs.2003.09.001

[58] Huber, B. et al. Interdisziplinäre Untersuchungen von Räuchergefäßen zur Rekonstruktion antiker Gerüche. *e-Forschungsberichte DAI* **2**, 120–125 (2018).

[59] Mathe, C., Archier, P., Nehme, L. & Vieillescazes, C. The study of Nabataean organic residues from Madâ’in Sâlih, ancient Hegra, by gas chromatography–mass spectrometry. *Archaeometry* **51**, 626–636 (2009).

[60] Betts, A. Ecstasy Meets Paleoethnobotany. in *The Routledge Companion to Ecstatic Experience in the Ancient World* 90–100 (Routledge, London, 2021). 10.4324/9781003041610-8.

[61] El-Rifaie, M. E. *Peganum harmala*: its use in certain dermatoses. *Int. J. Dermatol.* **19**, 221–222 (1980). 7399797 10.1111/j.1365-4362.1980.tb00305.x

[62] Abolhassanzadeh, Z., Aflaki, E., Yousefi, G. & Mohagheghzadeh, A. Randomized clinical trial of peganum oil for knee osteoarthritis. *J. Evid. Based Complement. Alter. Med* **20**, 126–131 (2015). 10.1177/2156587214566867 25654976

[63] Van Zeist, W. & De Roller, G. J. Plant remains from Maadi, a predynastic site in lower Egypt. *Veget Hist. Archaebot.* **2**, 1–14 (1993).

[64] Akhtar, M. F. et al. Appraisal of anti-arthritic and anti-inflammatory potential of FolkloricMedicinal plant *Peganum harmala*. *EMIDDT* **22**, 49–63 (2022). 10.2174/1871530321666210208211310 33563161

[65] Farzin, D. & Mansouri, N. Antidepressant-like effect of harmane and other β-carbolines in the mouse forced swim test. *Eur. Neuropsychopharmacol.* **16**, 324–328 (2006). 16183262 10.1016/j.euroneuro.2005.08.005

[66] Vahabzadeh, M., Banagozar Mohammadi, A. & Delirrad, M. Abortion induced by *Peganum harmala* ingestion in a pregnant woman: a case report and literature review. *IJMTFM*10.32598/ijmtfm.v9i3.25910 (2019).

[67] Berdai, M. A., Labib, S. & Harandou, M. *Peganum harmala* L. Intoxication in a pregnant woman. *Case Rep. Emerg. Med.* **2014**, 1–3 (2014). 10.1155/2014/783236 PMC4053228 24955262

[68] Panda, H. *Herbs Cultivation and Medicinal Uses*. (National Institute of Industrial Research, Delhi, 1999).

[69] Tanweer, A. J., Chand, N., Saddique, U., Bailey, C. A. & Khan, R. U. Antiparasitic effect of wild rue (*Peganum harmala* L.) against experimentally induced coccidiosis in broiler chicks. *Parasitol. Res.* **113**, 2951–2960 (2014). 24879014 10.1007/s00436-014-3957-y

[70] Mirzaei, M. Treatment of natural tropical theileriosis with the extract of the plant *Peganum harmala*. *Korean J. Parasitol.* **45**, 267 (2007). 18165708 10.3347/kjp.2007.45.4.267 PMC2532620

[71] Shulgin, A. T. & Shulgin, A. *Tihkal: The Continuation*. (Transform, Berkeley, 1997).

[72] Shahverdi, A. R. et al. Antimicrobial activity and main chemical composition of two smoke condensates from *Peganum harmala* seeds. *Z. Naturforsch. C* **60**, 707–710 (2005). 16320612 10.1515/znc-2005-9-1008

[73] Klapper, M. et al. Natural products from reconstructed bacterial genomes of the Middle and Upper Paleolithic. *Science* **380**, 619–624 (2023). 37141315 10.1126/science.adf5300

[74] Patalano, R., Zech, J. & Roberts, P. Leaf wax lipid extraction for archaeological applications. *Curr. Protoc. Plant Biol.* **5**, e20114 (2020). 32791571 10.1002/cppb.20114

[75] Sassoui, D., Seridi, R., Azin, K. & Usai, M. Evaluation of phytochemical constituents by GC-MS and antidepressant activity of *Peganum harmala* L. seeds extract. *Asian Pac. J. Trop. Dis.* **5**, 971–974 (2015).

[76] Farouk, L., Laroubi, A., Aboufatima, R., Benharref, A. & Chait, A. Evaluation of the analgesic effect of alkaloid extract of *Peganum harmala* L.: possible mechanisms involved. *J. Ethnopharmacol.* **115**, 449–454 (2008). 18054186 10.1016/j.jep.2007.10.014

