# Supplementary material for: Metabolic profiling reveals first evidence of fumigating drug plant Peganum harmala in Iron Age Arabia
Source: Commun Biol. 2025 May 23;8:720. doi: 10.1038/s42003-025-08096-7 (PMC12102341; doi:10.1038/s42003-025-08096-7)
Supplement: Supplementary file 1 — Supplementary Information [file 42003_2025_8096_MOESM1_ESM.pdf]

Supplementary Information

Huber, Barbara[1,2,3*] – Luciani, Marta [4,5*] – Abualhassan, Ahmed M. [6] – Giddings Vassão, Daniel [1,7] – Fernandes, Ricardo [1,8,9,10] - Devièse, Thibaut [2]

[1] Max Planck Institute of Geoanthropology, Department of Archaeology, Jena, Germany

[2] Centre de Recherche et d'Enseignement des Géosciences de l'Environnement, Aix-Marseille Université, CNRS, IRD, INRAE, Aix-en-Provence, France

[3] University of Tübingen, Institute for Archaeological Sciences, Tübingen, Germany

[4] University of Vienna, Department of Prehistoric and Historical Archaeology, Vienna, Austria

[5] University of Vienna, Human Evolution and Archaeological Sciences (HEAS), Vienna, Austria

[6] Heritage Commission, Ministry of Culture of the Kingdom of Saudi Arabia, Riyadh, Saudi Arabia

[7] Max Planck Institute for Chemical Ecology, Department of Biochemistry, Jena, Germany

[8] Department of Bioarchaeology, Faculty of Archaeology, University of Warsaw, Warsaw, Poland

[9] Faculty of Arts, Masaryk University, Brno, Czechia

[10] Climate Change and History Research Initiative, Princeton University, Princeton, United States of America

[*] corresponding authors

<u>Table of content:</u>

Additional Information on the archaeological contexts.......... 2

1. Area D 2

2. Area N 4

Supplementary Table 1.......... 7

Supplementary Table 2.......... 8

Additional Information on the archaeological contexts

The residues from three burners are analyzed in the paper: DA-QU_D-1, DA-QU_D-2 from censer QU.D.1167.F.6, discovered in Area D and samples DA-QU.N-1 and DA-QU.N-2 from burners QU.N.1253.F.1 and QU.N.2340.F.3 from Area N.

Both Area D and Area N are dwellings dated to the Middle Iron Age attested in two locations in the Residential Area in Qurayyah (Fig. 1(a)).

1. Area D

The large courtyard (Fig. S1) of a stone-walled dwelling (Building D-B1) contained ostrich eggshell fragments, beads, iron and alabaster artefacts, stone tools and bronze coins (Luciani & Alsaud 2018: 169-170)) and could be understood as both a residence and a jewelry-making facility. Through the stratigraphic sounding in its courtyard of we could identify a sequence of three major architectural phases (Buildings D-B1 – D-B3) spanning over six or seven centuries.

**Building**	**Phases**	**Dating**	**Location in Area D**
Building D-B1	1-4	Roman period?	eastern half of Area D
Building D-B2	1-14	4th to early 2nd century BCE	western half of Area D
Building D-B3	15-22+	8th – first half of the 6th century BCE	western half of Area D

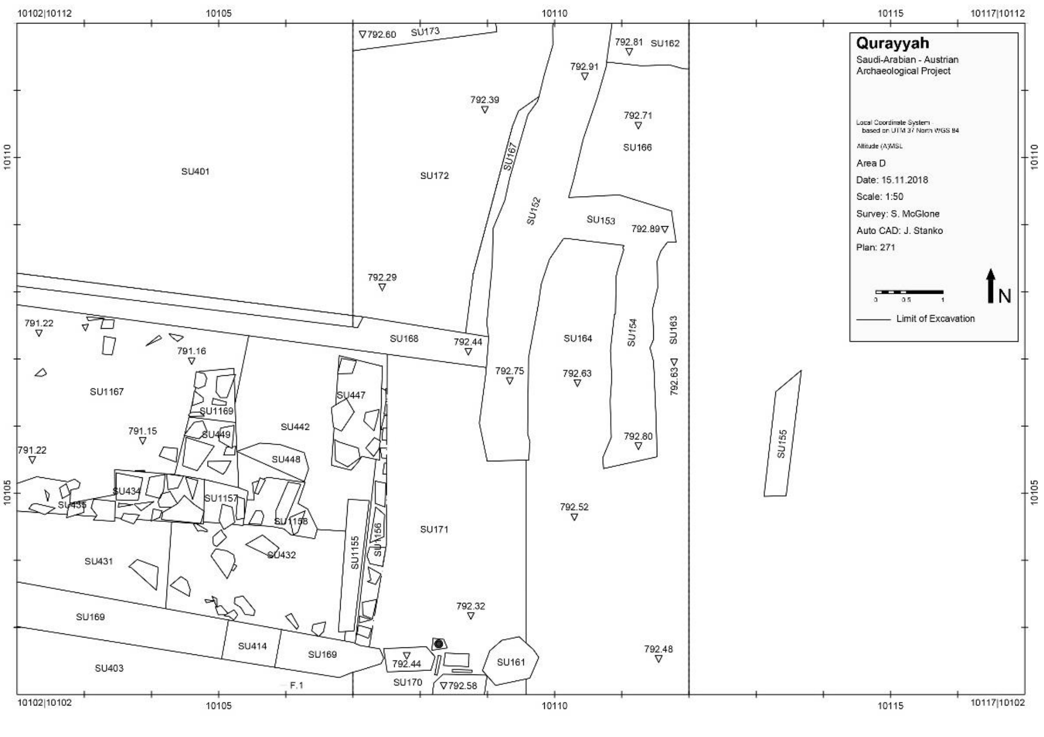

Fig. S1. Plan of Area D at the archaeological site of Qurayyah, with information about stratigraphic units (SU) and elevations.

From its elder, underlying unit Building D-B3, more specifically in Phase 17, we uncovered an open courtyard context. In situ, we found both the investigated censer QU.D.1167.F.6 (= samples DA-QU.D-1 and 2) and a painted vessel QU.D.1167.F.1 (Fig. S2). preserved on the same trodden floor. Additionally, we discovered an alabaster (calcite) bowl QU.D.1167.F5b along with two painted vessels: bowl QU.D.1167.F.1. and small bottle QU.D.1167.33+1172.1 (Luciani 2022b). The alabaster bowl may have been used for preparing the plants for fumigation. Interestingly, the vegetal patterns used to decorate both the bowl and the small bottle resemble the modern form of *Peganum Harmala* plants.

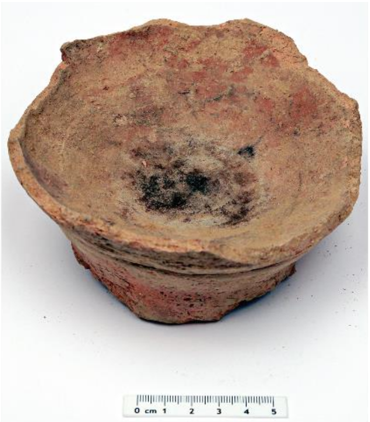

Censer QU.D.1167.F.6

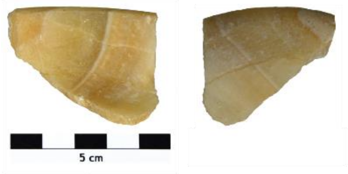

Alabaster (calcite) bowl QU.D.1167.F5b

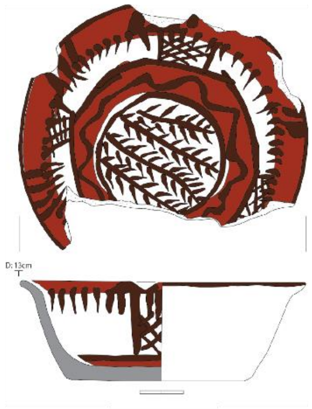

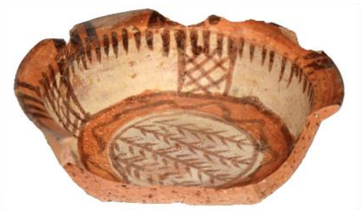

Pottery bowl QU.D.1167.F1

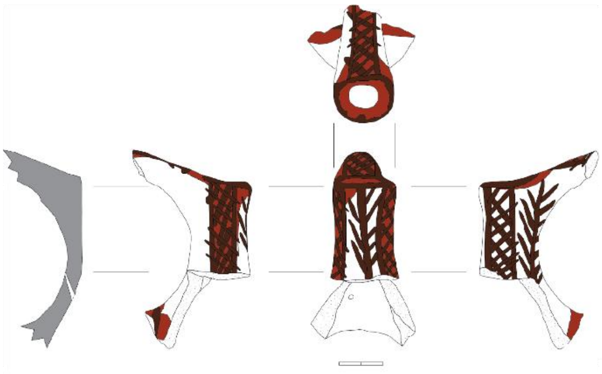

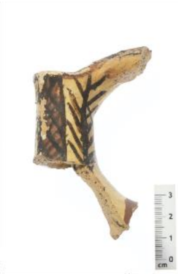

Pottery bottle QU.D.1172.1 / QU.D.1167.33

Fig. S2. Censer QU.D.1167.F.6 and associated archaeological objects from phase 17 of unit DB-3 in Area D. Note: Pottery drawings have a 2 cm scale bar beneath each drawing.

2. Area N

Localized north/northwest of Area D, the open area excavation here exposed ca. 80 square meters of a dwelling featuring an upper-class, stone architecture over multiple stories, including a stone-paved basement and several parallel rooms used for storage and food preparation. At least five 5 different building phases can be precisely distinguished from each other by stratigraphy, construction material and typology.

The Phase 5 (Fig. S3, light blue colour) basement (Room 1643) is the most ancient space constructed and used in this dwelling (Luciani 2022c). It was paved with large stone slabs and contained, besides the analysed censer QU.N.2340.F.3 (= sample DA-QU.N-2), several notable artifacts, including a number of fragmentary bichrome painted vessels (QU.N.2340.1, QU.N.2340.31, QU.N.2340.32, QU.N.2340.39), a fragment of an incised storage jar (QU.N.2340.2) and a small bottle (QU.N.2340.F.1) (Fig. S4; Kräuter 2024). These findings allow for several observations. The small bottle is significantly older, by almost a thousand years, than all the other objects and must be considered either a very well-kept heirloom or an ancient re-excavated object that was added to the assemblage. This possibly suggests that the cellar was a storage facility for important materials.

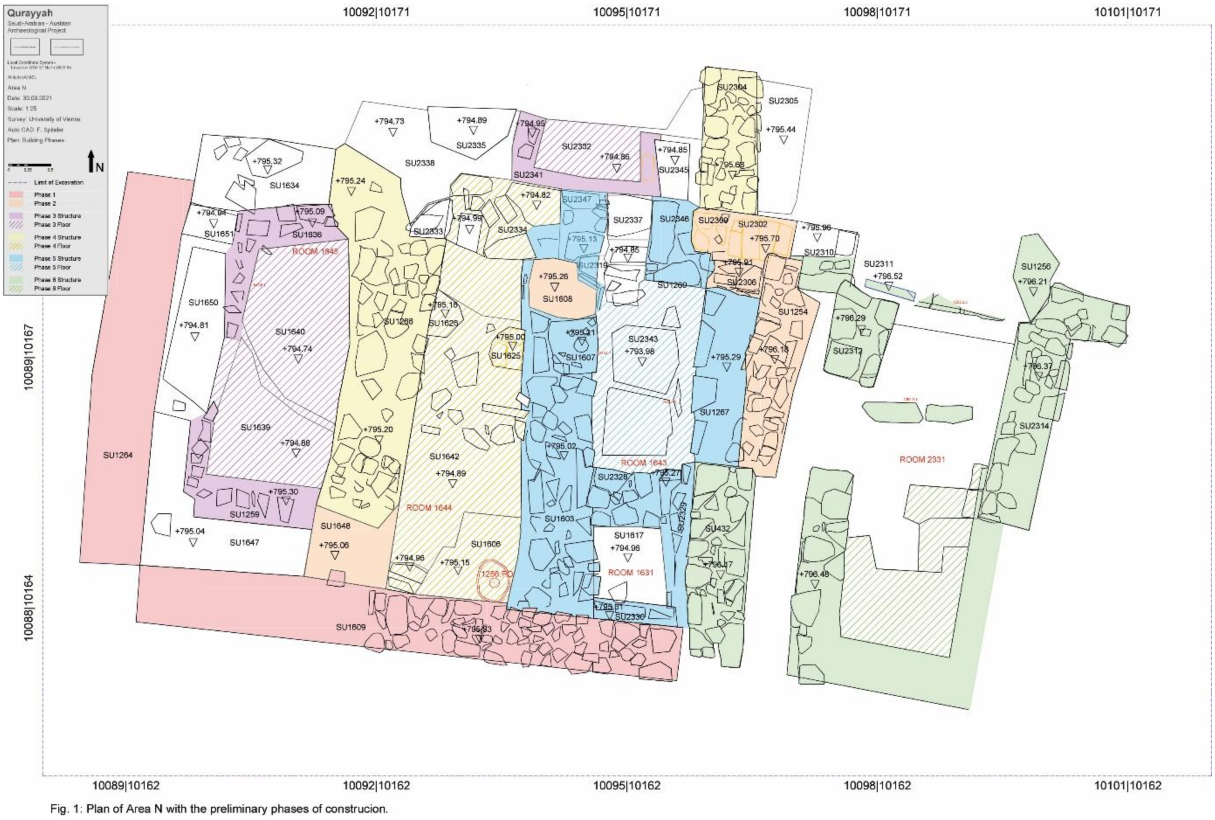

Fig. S3. Plan of Area N at the archaeological site of Qurayyah, showing an upper-class stone building with 5 building phases.

The bichrome painted pottery exhibits a style similar to the pottery found in Area D, but two vessels are particularly remarkable: QU.N.2340.1 is a typical cup with the bichrome painted representation of a dromedary camel, the quintessential pack animal domesticated for the transport of South Arabian aromata to the Mediterranean. The other vessel, a flat bowl QU.N.2340.39, also bichrome painted, shows clear signs of having been reused –when already chipped– as censer. We have not yet sampled these residues, but both these vessels could be symbolically and/or factually linked with the import and use of fragrances.

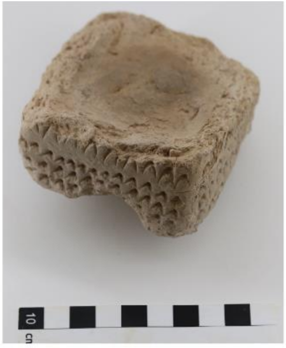

Censer QU.N.2340.F.3

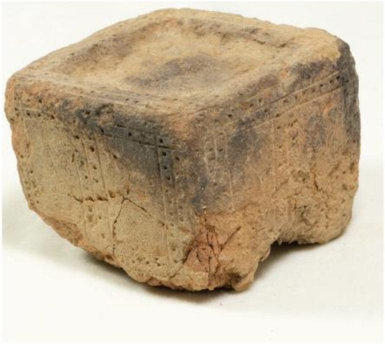

Censer QU.N.1253.F.1

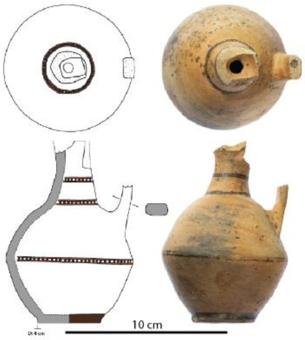

Bottle QU.N.2340.F1

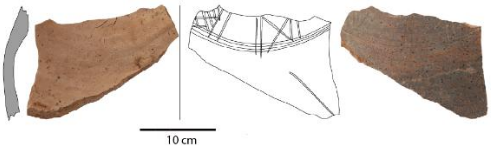

Storage jar QU.N.2340.2

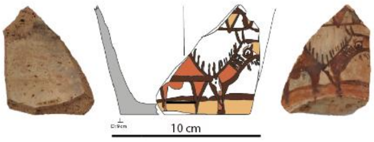

QU.N.2340.1

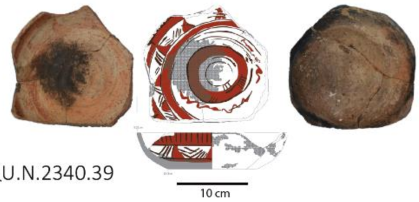

QU.N.2340.39

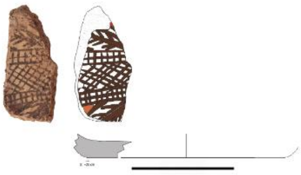

QU.N.2340.31 10 cm

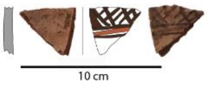

QU.N.2340.32

Fig. S4. Censers from Area N and associated archaeological objects from the upper-class dwelling. Note: Pottery drawings are shown at varying scales, with a 10 cm scale bar beneath each drawing.

In the youngest phases of use of the building Phase 1 (Fig. S3, pink colour), at the southern end of Room 1644, an area used over several phases for food preparation and cooking in a clay oven (*tannur* QU.N.1256), finds include besides the investigated square burner QU.N.1253.F.1 (= sample DA-QU.N-1 Fig. S4;), an Egyptian rectangular seal amulet and a large, millefiori glass bead (Luciani 2022c, 38-39). We do not have radiometric measurements of organic remains in Area N. However, in view of the general resemblance of its pottery to the one found in Area D, we can estimate the life of the Area N dwelling to encompass the "Hallstatt Plateau" period, i.e. the 8th – 6th century BCE. However, gauging the chronological difference between Phase 5 and Phase 1 remains unsubstantiated.

References:

Luciani, M. 2022b, *Area R, Qurayyah 2018. Report on the Fourth Season of the Joint Saudi Arabian-Austrian Archaeological Project* (eds. Luciani, M. & Abualhassan A.M.) vol. 32 33–38 (2022).

Luciani, M. 2022c, *Area N, Qurayyah 2018. Report on the Fourth Season of the Joint Saudi Arabian-Austrian Archaeological Project* (eds. Luciani, M. & Abualhassan A.M.) vol. 32 38–39 (2022).

Luciani, M. & Alsaud, A. S. 2018, The new archaeological joint project on the site of Qurayyah, north-west Arabia: results of the first two excavation seasons. Proceedings of the Seminar for Arabian Studies 48 (2018): 165–183.

Kräuter, R.G. 2024 *The Iron Age Pottery of Qurayyah*. Unpublished Master's Thesis, Department of Prehistoric and Historical Archaeology, University of Vienna.

Supplementary Table 1

Table S1. List of radiocarbon dates from Area D at the oasis of Qurayyah, Nordwest Arabia measured at the Center for Applied Isotope Studies, University of Atlanta, Georgia. Carbon stable isotope ratios (AMS values) are presented in ‰ relative to Vienna PeeDee Belemnite. Radiocarbon concentrations are reported as pMC (Percent Modern Carbon). The uncalibrated ^{14}C ages were calibrated using OxCal v4.4.4 (Bronk Ramsey 2021, Reimer *et al.* 2020). All dates in this phase point to the so-called *Hallstatt Plateau* interval: 8th – 6th centuries cal BCE.

Lab code	Sample ID (QU.Area.SU)	Material	$\delta^{13}C$ ‰	pMC	^{14}C age (yrs BP)	Calibrated ^{14}C ages (calBCE 95,4%)	Deposits' sequence in Qurayyah
UGAMS #46400	QU.D.1167.S.5	Wood, Tamarisk branch	-25,37	73,14 ± 0,19	2510 ± 20	776-734 calBCE (20,3%)<br>696-662 calBCE (18,3%)<br>650-545 calBCE (56,9%)	Area D: Phase 17 – house in Residential Area
UGAMS #46401	QU.D.1583.S.2	Wood, Tamarisk branch	-26,06	73,26 ± 0,19	2500 ± 20	773-725 calBCE (18,7%)<br>703-662 calBCE (18,5%)<br>651-544 calBCE (58,3%)	Area D: Phase 18 – house in Residential Area
UGAMS #46402	QU.D.1600.S.2	Wood, Tamarisk branch	-24,79	73,26 ± 0,19	2500 ± 20	773-725 calBCE (18,7%)<br>703-662 calBCE (18,5%)<br>651-544 calBCE (58,3%)	Area D: Phase 20 – house in Residential Area

Supplementary Table 2

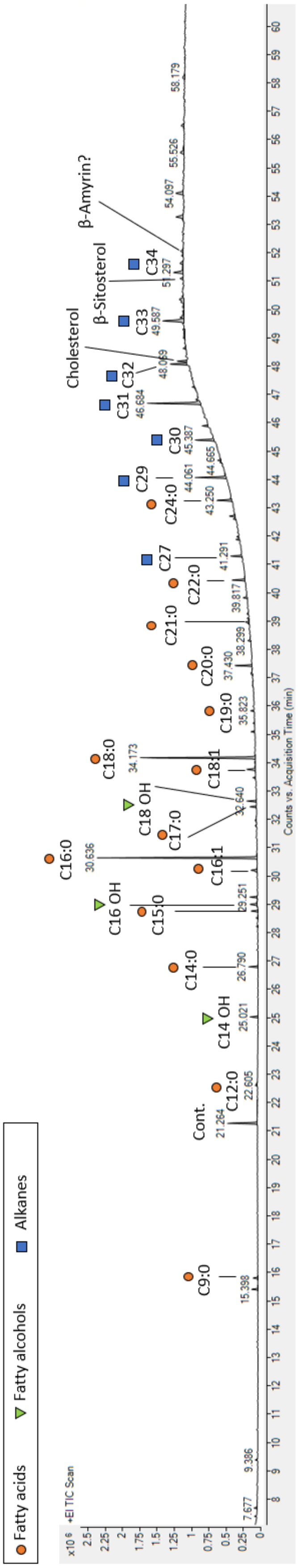

Supplementary Table S2. Total ion current (TIC) chromatogram obtained by GC-MS of sample DA-QU_N-1. The orange symbol shows fatty acids, n:0 = saturated FA and n:1 = unsaturated FA; the green symbol displays the distribution of fatty alcohols and the blue symbol shows *n*-alkanes with corresponding carbon numbers.

Additional analysis using GC-MS identified a range of lipids. The lipid profile was dominated by high abundances of saturated, even-carbon-numbered straight-chain fatty acids, primarily palmitic acid (C16:0) and stearic acid (C18:0). These free fatty acids are common degradation products of lipidic substances and may indicate contributions from plant oils or animal/human fats[1,2]. The sample also exhibited the short-chain fatty acid C9:0, which is recognized as a degradation product formed through oxidation[3,4]. Additionally, we detected n-alkanes with a slight odd-over-even predominance, with C31 being the most abundant. Such odd-over-even dominance is characteristic of epicuticular plant waxes. In line with the LC-MS/MS analysis, small amounts of cholesterol, β-sitosterol, and potentially β-amyrin were also detected. In summary, the composition of fatty acids in the sample, particularly the high proportion of C16:0, and the n-alkane profile, characteristic of plant waxes, suggests a plant-based origin. However, more specific details regarding the plant species cannot be determined based on these results alone.

References:

1. Whelton, H. L. *et al.* A call for caution in the analysis of lipids and other small biomolecules from archaeological contexts. *J. Archaeol. Sci.* **132**, 105397 (2021).

2. Roffet-Salque, M. *et al.* From the inside out: Upscaling organic residue analyses of archaeological ceramics. *J. Archaeol. Sci. Rep.* **16**, 627–640 (2017).

3. Huber, B. *et al.* Biomolecular characterization of 3500-year-old ancient Egyptian mummification balms from the Valley of the Kings. *Sci. Rep.* **13**, 12477 (2023).

4. Łucejko, J., Connan, J., Orsini, S., Ribechini, E. & Modugno, F. Chemical analyses of Egyptian mummification balms and organic residues from storage jars dated from the Old Kingdom to the Copto-Byzantine period. *J. Archaeol. Sci.* **85**, 1–12 (2017).
