# Supplementary material for: Metabolic profiling reveals first evidence of fumigating drug plant Peganum harmala in Iron Age Arabia
Source: Commun Biol. 2025 May 23;8:720. doi: 10.1038/s42003-025-08096-7 (PMC12102341; doi:10.1038/s42003-025-08096-7)
Supplement: Supplementary file 2 — Description of Additional Supplementary Files [file 42003_2025_8096_MOESM2_ESM.pdf]

Description of Additional Supplementary Files

File name: Supplementary Data 1-4

Description: MRM chromatograms of archaeological samples including analytical standards

File name: Supplementary Data 5

Description: MRM Parameters
