# Supplementary material for: Metabolic profiling reveals first evidence of fumigating drug plant Peganum harmala in Iron Age Arabia
Source: Commun Biol. 2025 May 23;8:720. doi: 10.1038/s42003-025-08096-7 (PMC12102341; doi:10.1038/s42003-025-08096-7)
Supplement: Supplementary file 3 — Supplementary Data 1-4 [file 42003_2025_8096_MOESM3_ESM.docx]

# Supplementary Data

**Supplementary Data 1.** List of compounds present in the archaeological sample DA-QU_D-1 (right column) compared to authentic analytical standards (left column). The MRM chromatograms show peaks of specific precursor and product ion pairs as well as the different collision energies applied. For each compound, at least three distinct MRM transitions were monitored (with the exception of coumarin). The presence of a specific compound in the archaeological sample was confirmed when the observed transitions and chromatographic retention times (displayed in min) matched those of the analytical standards analyzed under identical conditions.

| **Standards** | **Sample DA-QU_D-1** |
| --- | --- |
| Cholesterol   |  |
| Campesterol   |  |
| β-Sitosterol   |  |
| Stigmasterol   |  |
| Sitostanol   |  |
| Harmine   |  |
| Harmane  **** |  |

**Supplementary Data 2.** List of compounds present in the archaeological sample DA-QU_D-2 (right column) compared to authentic analytical standards (left column). The MRM chromatograms show peaks of specific precursor and product ion pairs as well as the different collision energies applied. For each compound, at least three distinct MRM transitions were monitored (with the exception of coumarin). The presence of a specific compound in the archaeological sample was confirmed when the observed transitions and chromatographic retention times (displayed in min) matched those of the analytical standards analyzed under identical conditions.

| **Standards** | **Sample DA-QU_D-2** |
| --- | --- |
| Cholesterol   |  |
| Campesterol   |  |
| β-Sitosterol   |  |
| Stigmasterol   |  |
| Sitostanol   |  |
| Coumarin   |  |
| Harmine   |  |
| Harmane  **** |  |

**Supplementary Data 3.** List of compounds present in the archaeological sample DA-QU_N-1 (right column) compared to authentic analytical standards (left column). The MRM chromatograms show peaks of specific precursor and product ion pairs as well as the different collision energies applied. For each compound, at least three distinct MRM transitions were monitored. The presence of a specific compound in the archaeological sample was confirmed when the observed transitions and chromatographic retention times (displayed in min) precisely matched those of the analytical standards analyzed under identical conditions.

| **Standards** | **Sample DA-QU_N-1** |
| --- | --- |
| Cholesterol   |  |
| β-Sitosterol   |  |
| α-Amyrin   |  |
| β-amyrin   |  |
| Harmine   |  |
| Harmane  **** |  |

**Supplementary Data 4.** List of compounds present in the archaeological sample DA-QU_N-2 (right column) compared to authentic analytical standards (left column). The MRM chromatograms show peaks of specific precursor and product ion pairs as well as the different collision energies applied. For each compound, at least three distinct MRM transitions were monitored. The presence of a specific compound in the archaeological sample was confirmed when the observed transitions and chromatographic retention times (displayed in min) matched those of the analytical standards analyzed under identical conditions.

| **Standards** | **Sample DA-QU_N-2** |
| --- | --- |
| Cholesterol   |  |
| β-Sitosterol   |  |
| α-Amyrin   |  |
| β -Amyrin   |  |
