# Supplementary material for: Metabolic profiling reveals first evidence of fumigating drug plant Peganum harmala in Iron Age Arabia
Source: Commun Biol. 2025 May 23;8:720. doi: 10.1038/s42003-025-08096-7 (PMC12102341; doi:10.1038/s42003-025-08096-7)
Supplement: Supplementary file 5 — Reporting summary [file 42003_2025_8096_MOESM5_ESM.pdf]

nature portfolio

Corresponding author(s): Barbara Huber, Marta Luciani

Last updated by author(s): 01.04.2025

Reporting Summary

Nature Portfolio wishes to improve the reproducibility of the work that we publish. This form provides structure for consistency and transparency in reporting. For further information on Nature Portfolio policies, see our Editorial Policies and the Editorial Policy Checklist.

Statistics

For all statistical analyses, confirm that the following items are present in the figure legend, table legend, main text, or Methods section.

n/a	Confirmed	
☒	☐	The exact sample size (*n*) for each experimental group/condition, given as a discrete number and unit of measurement
☒	☐	A statement on whether measurements were taken from distinct samples or whether the same sample was measured repeatedly
☒	☐	The statistical test(s) used AND whether they are one- or two-sided<br>*Only common tests should be described solely by name; describe more complex techniques in the Methods section.*
☒	☐	A description of all covariates tested
☒	☐	A description of any assumptions or corrections, such as tests of normality and adjustment for multiple comparisons
☒	☐	A full description of the statistical parameters including central tendency (e.g. means) or other basic estimates (e.g. regression coefficient) AND variation (e.g. standard deviation) or associated estimates of uncertainty (e.g. confidence intervals)
☒	☐	For null hypothesis testing, the test statistic (e.g. *F*, *t*, *r*) with confidence intervals, effect sizes, degrees of freedom and *P* value noted<br>*Give P values as exact values whenever suitable.*
☒	☐	For Bayesian analysis, information on the choice of priors and Markov chain Monte Carlo settings
☒	☐	For hierarchical and complex designs, identification of the appropriate level for tests and full reporting of outcomes
☒	☐	Estimates of effect sizes (e.g. Cohen's *d*, Pearson's *r*), indicating how they were calculated

Our web collection on statistics for biologists contains articles on many of the points above.

Software and code

Policy information about availability of computer code

Data collection	No code was used for data collection.
Data analysis	LabSolutions software (Shimadzu, Kyoto, Japan) was used for data acquisition and analysis.

For manuscripts utilizing custom algorithms or software that are central to the research but not yet described in published literature, software must be made available to editors and reviewers. We strongly encourage code deposition in a community repository (e.g. GitHub). See the Nature Portfolio guidelines for submitting code & software for further information.

Data

Policy information about availability of data

All manuscripts must include a data availability statement. This statement should provide the following information, where applicable:

- Accession codes, unique identifiers, or web links for publicly available datasets
- A description of any restrictions on data availability
- For clinical datasets or third party data, please ensure that the statement adheres to our policy

All data generated or analyzed during this study are included in this published article and its supplementary information files.

Research involving human participants, their data, or biological material

Policy information about studies with human participants or human data. See also policy information about sex, gender (identity/presentation), and sexual orientation and race, ethnicity and racism.

Reporting on sex and gender	n/a
Reporting on race, ethnicity, or other socially relevant groupings	n/a
Population characteristics	n/a
Recruitment	n/a
Ethics oversight	n/a

Note that full information on the approval of the study protocol must also be provided in the manuscript.

Field-specific reporting

Please select the one below that is the best fit for your research. If you are not sure, read the appropriate sections before making your selection.

☐ Life sciences ☒ Behavioural & social sciences ☐ Ecological, evolutionary & environmental sciences

For a reference copy of the document with all sections, see nature.com/documents/nr-reporting-summary-flat.pdf

Behavioural & social sciences study design

All studies must disclose on these points even when the disclosure is negative.

Study description	An interdisciplinary study combining archaeological research with metabolic profiling of organic residues was conducted. Residues were sampled from fumigation devices found at the ancient oasis of Qurayyah. The samples were analyzed using liquid chromatography-tandem mass spectrometry (LC-MS/MS) and subjected to qualitative evaluation to identify organic compounds, with a focus on their potential medicinal and psychoactive ingredients and their cultural or functional significance.
Research sample	Four samples of organic remains from the fumigation devices were taken. All samples originate from the residential area of the archaeological site: two from Area D, an Iron Age residence, and two from Area N, an elite dwelling from the same period.
Sampling strategy	All available fumigation devices from the residential area at the oasis of Qurayyah have been selected and sampled for analysis. However, as this is an ongoing excavation, additional devices may be discovered during future campaigns. Sampling followed the commonly accepted protocol for extracting organic residues impregnated in archaeological ceramics. Prior to sampling, the uppermost inner layer of each fumigation device was carefully abraded to remove surface contaminants. Visible incrustations were excised with a scalpel, and deeper layers were sampled using a Dremel 200 drill with tungsten drill bits, extracting 1-2 grams of powder from the clay matrix. Drill bits were cleaned between samplings to prevent cross-contamination.
Data collection	The fumigation devices were excavated at the ancient oasis of Qurayyah by a joint Saudi-Austrian mission, financed by the Heritage Commission of the Ministry of Culture, the Augustus Foundation and the Faculty of Historical and Cultural Studies of the University of Vienna. Sampling was conducted at the archaeological site post-excavation and within the laboratories of the Max Planck Institute of Geoantropology in Jena, Germany. Sample extraction, LC-MS/MS analysis and data analysis took also place at the Max Planck Institute of Geoantropology. Data were recorded in Multiple Reaction Monitoring (MRM) mode. MRM is a targeted mass spectrometry technique, which facilitates the monitoring of specific precursor and product ion pairs, significantly enhancing specificity and sensitivity of the analysis. This proves particularly advantageous for detecting compounds that are low in abundance in archaeological contexts.
Timing	The fumigation devices were excavated during the 2018 and 2021 campaigns at Qurayyah. Sampling at Qurayyah (Area N) occurred in September 2021, while the sampling from Area D and the subsequent analysis took place in June 2023.
Data exclusions	No data were excluded from the analysis.
Non-participation	n/a
Randomization	n/a

Reporting for specific materials, systems and methods

We require information from authors about some types of materials, experimental systems and methods used in many studies. Here, indicate whether each material, system or method listed is relevant to your study. If you are not sure if a list item applies to your research, read the appropriate section before selecting a response.

Materials & experimental systems

n/a	Involved in the study
☒	☐ Antibodies
☒	☐ Eukaryotic cell lines
☐	☒ Palaeontology and archaeology
☒	☐ Animals and other organisms
☒	☐ Clinical data
☒	☐ Dual use research of concern
☒	☐ Plants

Methods

n/a	Involved in the study
☒	☐ ChIP-seq
☒	☐ Flow cytometry
☒	☐ MRI-based neuroimaging

Palaeontology and Archaeology

Specimen provenance

The organic residue samples come from the archaeological site Qurayyah in Saudi Arabia. The excavation permit for research in Qurayyah as well as for analysis was issued by the former Saudi Commission for Tourism and Antiquities (now Heritage Commission of the Ministry of Culture). The representative of the Heritage Commission, Mr. Abualhassan, is a co-author of the paper.

Specimen deposition

The fumigation devices are currently stored at the excavation site in Saudi Arabia as well as at the University of Vienna.

Dating methods

Radiocarbon dates from Area D at the oasis of Qurayyah were measured at the Center for Applied Isotope Studies, University of Atlanta, Georgia. Carbon stable isotope ratios (AMS values) are presented in ‰ relative to Vienna PeeDee Belemnite. Radiocarbon concentrations are reported as pMC (Percent Modern Carbon). The uncalibrated 14C ages were calibrated using OxCal v4.4.4 (Bronk Ramsey 2021, Reimer et al. 2020).

☒ Tick this box to confirm that the raw and calibrated dates are available in the paper or in Supplementary Information.

Ethics oversight

No ethical approval or guidance was required. We followed accepted and published protocols for organic residue analysis in archaeological science.

Note that full information on the approval of the study protocol must also be provided in the manuscript.

Plants

Seed stocks

n/a

Novel plant genotypes

n/a

Authentication

n/a
